# Outcomes of permanent pacemakers and implantable cardioverter-defibrillators in an adult congenital heart disease population

**DOI:** 10.1016/j.ijcchd.2023.100490

**Published:** 2023-12-18

**Authors:** Jason Chami, Benjamin M. Moore, Calum Nicholson, Rachael Cordina, David Baker, David S. Celermajer

**Affiliations:** a Sydney Medical School, The University of Sydney, Camperdown, Australia; b Heart Research Institute, Newtown, Australia; c University of British Columbia, Vancouver, BC, Canada; d Royal Prince Alfred Hospital, Camperdown, Australia

**Keywords:** Adult congenital heart disease, Arrhythmia, Implantable devices, Pacemakers, Implantable cardioverter-defibrillators

## Abstract

**Background:**

Brady- and tachyarrhythmias commonly complicate adult congenital heart disease (ACHD). Permanent pacemakers (PPMs) or implantable cardioverter–defibrillators (ICDs) are often utilised to prevent morbidity or mortality related to arrhythmia, but can also be associated with significant morbidity themselves.

**Methods:**

We analysed outcomes from patients in our comprehensive ACHD database who were seen at least twice since 2000 and once since 2018. Of 1953 ACHD patients, 134 had a PPM and 78 had an ICD (47 for primary and 31 for secondary prevention).

**Results:**

For PPM patients, 41% had a pacing percentage below 33%, 13% had 33–66%, and 46% had above 66%. One fifth required PPM upgrade, most to cardiac resynchronisation therapy, the rest to ICD. There were 33 appropriate ICD shocks in 15 patients (19%) and 34 inappropriate shocks in 13 patients (17%) over a median follow up of 4.6 years (IQR 0.9–8.3 years). Anti-tachycardia pacing was delivered appropriately for 28% of patients and inappropriately for 9%.

Apart from inappropriate therapy, one third of PPM and ICD patients had other device-related complications. Acute PPM complications included lead dysfunction requiring revision (2%), pneumothorax (2%), pleural effusion (2%) and pocket infection (2%). ICDs were also acutely complicated by lead dysfunction (4%) as well as pocket hematoma (3%). The most common long-term complication overall was lead dysfunction, affecting one sixth of both PPM and ICD patients. Finally, the rate of device insertion increased significantly with disease severity.

**Conclusions:**

Anti-arrhythmic devices can be lifesaving in ACHD patients, but inappropriate therapy and device-related complications are very common.

## 1 Introduction

Congenital heart disease (CHD) is the most common type of birth defect, affecting hundreds of thousands of children (and now adults) worldwide [1]. CHDs are often multiple, complex and comorbid, necessitating increasingly specialised care and monitoring across their lifespan, as a result of the markedly improved prognosis of CHD patients over the last 20 years [2,3]. As CHD patients live longer lives, the burden of arrhythmia in their structurally abnormal hearts has become increasingly apparent [4].

This increased risk of brady- and tachyarrhythmia is multifactorial [4], and can be related to structural abnormalities (e.g. atrioventricular block in transposition of the great arteries; TGA) or as a result of previous surgery or abnormal cardiac pressures (e.g. following the surgical repair of Tetralogy of Fallot; TOF). The management of cardiac arrhythmia in CHD is complex, regularly involving medical therapy, repeat electrophysiological studies with radiofrequency ablation, permanent pacemakers (PPMs) or implantable cardioverter-defibrillators (ICDs). Despite their efficacy [5,6], these implantable devices can be associated with significant morbidity, including surgical complications, inappropriate therapy and cardiac perforation [7].

PPMs and ICDs have been available for decades and have well-characterised risks in the common contexts of atrioventricular block after myocardial infarction or following cardiac arrest due to ventricular tachycardia/fibrillation. Most recipients are elderly and have structurally “normally-connected” hearts. There is reason to believe, however, that the risk profile of such implantable anti-arrhythmic devices will be altered in Adult CHD (ACHD) patients [8] as a result of younger age, structurally complex hearts and comorbidities predisposing towards early surgical complications [9] and/or long-term device failure [10]. The therapeutic benefit of these devices in the face of such high reported rates of complications has not been adequately characterised [11]; after all, because of the rarity of these lesions, the decision to implant is consensus-based and extrapolated from data on acquired heart disease rather than on large, robust randomised controlled trials in the ACHD population. It is essential that the risks and benefits of PPMs and ICDs are accurately characterised in the unique setting of ACHD, so therapy can be evidence-based, and patients can be accurately informed.

In view of the rarity of most congenital cardiac lesions and the comparatively recent emergence of the subspeciality of adult congenital electrophysiology, finding a data source large and comprehensive enough to make statistically significant claims about the outcomes of implantable anti-arrhythmic devices in ACHD is a challenge. The Royal Prince Alfred Hospital (RPAH) ACHD database is uniquely placed to fill this gap. Established over 10 years ago and updated every week, this database, representing the only quaternary ACHD referral centre in the state of New South Wales, Australia, collects information from hundreds of ACHD patients across the full socioeconomic, age and geographic spectrum. We therefore decided to analyse this database to investigate the outcomes of PPMs and ICDs in ACHD.

This study aims to examine the outcomes of PPMs and ICDs in all ACHD patients seen at RPAH at least twice since 2000 who were also seen at least once since 2018, to ensure that their medical records would be available for manual search. Outcome variables of interest are device-related complications, delivery of appropriate and inappropriate therapies, device-related parameters and survival. We hypothesise that ACHD patients will have an altered risk profile for PPMs and ICDs as a result of their unique predisposing factors.

## 2 Methods

### 2.1 Ethics and consent

The RPAH ACHD database is approved for retrospective research by the Human Research Ethics Committee and Royal Prince Alfred Hospital (X18-0189 & 2019/STE08327). This project has a waiver of consent to collect retrospective data.

### 2.2 Data collection

The RPAH ACHD database was used to retrospectively select all patients who had been seen at least twice since 2000 and at least once since 2018. The year 2000 was when basic patient information (including the presence of antiarrhythmic devices) began to be digitised, allowing us to electronically select patients for further manual search. Physical records from patients not seen in the last 5 years are often relocated, so we chose to focus on patients seen in the last 5 years so we could be confident of finding their records. We extracted their patient ID, age, sex, and diagnosis summary. From this list, physical patient records were collected from RPAH, as well as from the rooms of Professor David Celermajer and A/Prof Rachael Cordina. For all patients in the database with anti-arrhythmic devices, the decision to insert the device was made by an expert ACHD physician. The insertions themselves were carried out by specialist physicians or surgeons with knowledge of ACHD anatomy and physiology.

For PPM patients we extracted date of last follow up, date of implant, type of device (atrial only, ventricular only or dual), acute complications (within 30 days of implant), long-term complications, generator changes, ventricular pacing percentage at last device interrogation, any device upgrades (to ICD or cardiac resynchronisation therapy; CRT) and death.

For ICD patients, we extracted date of last follow up, indication for insertion (primary or secondary prevention), date of implant, device type (single chamber, dual chamber, CRT or subcutaneous), acute and long-term complications, inappropriate/appropriate shocks/anti-tachycardia pacing and their causes, generator changes and death. Although ICDs also have pacing capability, ventricular pacing information was only available for half of ICD patients, and was thus not analysed to ensure validity.

The RPAH ACHD database maintains up-to-date mortality statistics via linkage to the Australian National Death Index (NDI). Vital status was extracted and linked for all eligible PPM and ICD patients.

### 2.3 Data analysis and statistical methods

For all patients, disease severity according to the 2020 European Society of Cardiology guidelines [12] was calculated from diagnoses using a previously described algorithm developed in-house [13]. Kaplan–Meier curves were generated for survival and for freedom from complication; curves were compared using log-rank tests. *T*-tests compared the proportion of primary and secondary prevention ICD patients who received any appropriate therapy, as well as the difference in complication rate between patients with mild and severe disease. Chi-squared tests were performed to test the hypothesis that implantable anti-arrhythmic device prevalence increased with disease severity.

## 3 Results

Of 1953 patients in the RPAH ACHD database seen at least twice since 2000 and once since 2018, we found 133 with a PPM (7%) and 78 with an ICD (4%). Patient characteristics are summarised in Table 1.

**Table 1:** Patient characteristics of 133 permanent pacemaker (PPM) patients and 78 implantable cardioverter-defibrillator (ICD) patients, selected from 1953 patients seen at the Royal Prince Alfred Hospital Adult Congenital Heart Disease Clinic at least twice since 2000 and once since 2018.

	PPM, N = 133^a^	ICD, N = 78^a^
**Gender**		
Male	68 (51%)	47 (60%)
Female	65 (49%)	31 (40%)
**Age at Insertion**	32 (15, 44)	39 (29, 48)
**Diagnosis**		
Tetralogy of Fallot	17 (13%)	39 (50%)
Transposition of the Great Arteries (Palliated)	26 (20%)	9 (12%)
Fontan (Univentricular Heart)	25 (19%)	2 (2.6%)
Other Complex Congenital Heart Disease	14 (11%)	11 (14%)
Congenitally Corrected Transposition of the Great Arteries	14 (11%)	3 (3.8%)
Atrioventricular Septal Defect	10 (7.5%)	1 (1.3%)
Coarctation of the Aorta	5 (3.8%)	6 (7.7%)
Ebstein's Anomaly	5 (3.8%)	5 (6.4%)
Aortic Stenosis	7 (5.3%)	2 (2.6%)
Atrial Septal Defect	4 (3.0%)	0 (0%)
Ventricular Septal Defect	4 (3.0%)	0 (0%)
Eisenmenger Syndrome	2 (1.5%)	0 (0%)
**Follow Up Years**	9 (4, 21)	5 (3, 10)
**Device Subtype**		
Dual-Chamber ICD	–	48 (62%)
Single-Chamber ICD	–	24 (31%)
Cardiac Resynchronisation Therapy-Defibrillator	–	5 (6.4%)
Subcutaneous ICD	–	1 (1.3%)
Dual-Chamber PPM	93 (70%)	–
Ventricular PPM	27 (20%)	–
Atrial PPM	13 (9.8%)	–
**ICD Indication**		
Primary Prevention	–	47 (60%)
Secondary Prevention	–	31 (40%)

a n (%); Median (IQR).

### 3.1 PPM

**Fig. 1:**
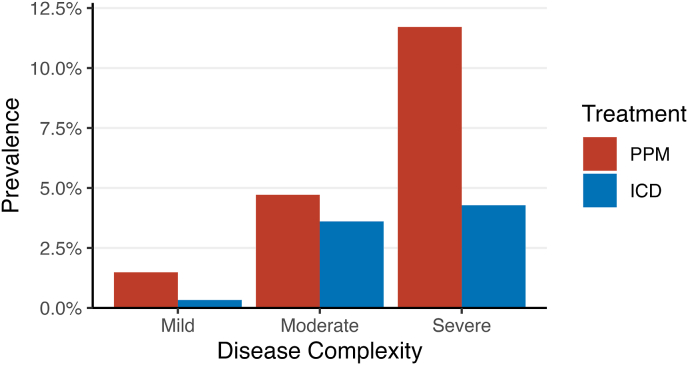
Prevalence of permanent pacemakers (PPMs) and implantable cardioverter-defibrillators (ICDs) among all Royal Prince Alfred Hospital adult congenital heart disease patients seen at least twice since 2000 who have also been seen at least once since 2018, stratified by disease severity according to the 2020 European Society of Cardiology guidelines [12] using an algorithm developed in-house [13].

Overall, 26 PPMs were found out of 217 eligible patients who had TGA treated with an atrial switch (12%), 25 out of 82 patients with a Fontan circulation (30%), 17 out of 332 patients with TOF (5%), 14 out of 54 patients with congenitally corrected TGA (26%) and 10 out of 107 patients with an atrioventricular septal defect (9%); 41 had other CHD diagnoses. Median age at implantation was 32 years, and the cohort was 51% male. Two thirds of all PPMs were dual-chamber (93 patients, 69%), 27 (20%) were ventricular, and 13 (10%) were atrial. The rate of PPM implantation increased significantly with disease severity (χ^2^-test, p < 0.001, Fig. 1).

Of the 25 patients with a Fontan circulation, 17 had a dual-chamber PPM with epicardial leads, 2 had a dual-chamber PPM with one endocardial atrial lead and one epicardial ventricular lead, 4 had a single-chamber PPM with one epicardial ventricular lead only, and 2 had a single-chamber PPM with one endocardial atrial pacing lead only.

**Fig. 2:**
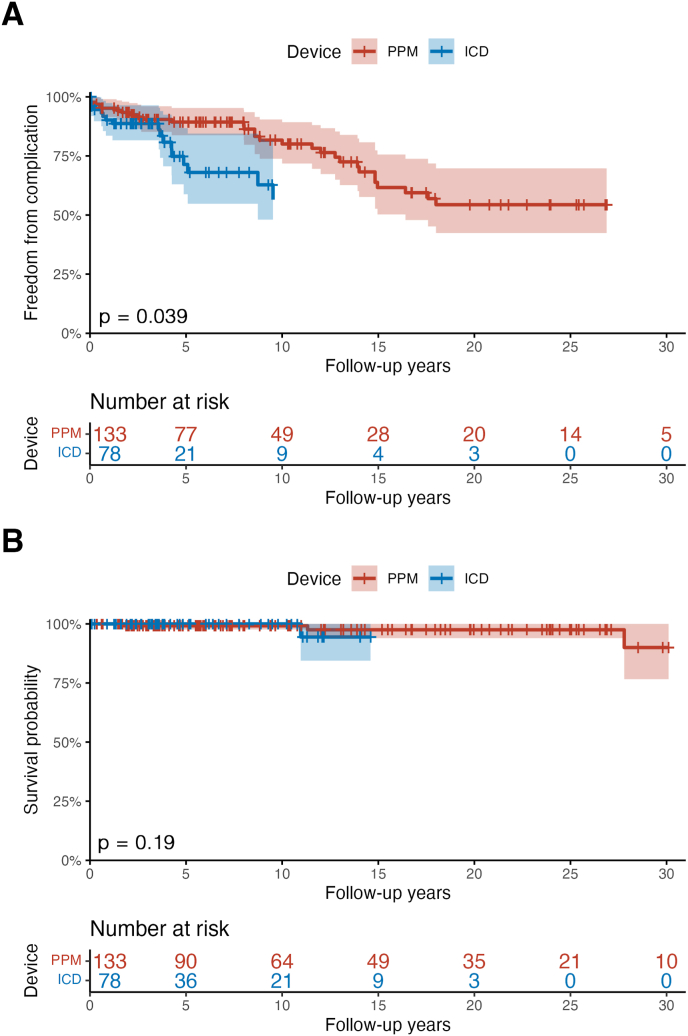
Kaplan–Meier curves showing (A) freedom from complications including inappropriate therapy, and (B) survival, in years after device implantation. Curves are truncated at n = 10 patients. P-values calculated using the log-rank method.

Over a median 8.8 year follow up period (IQR 0.25–17.4 years), all-cause mortality was 3% (Fig. 2B), none of which was attributable to the device itself. Ventricular pacing was below 33% in 41% of applicable PPM patients, between 33 and 66% in 13% of patients, and above 66% in 46% of patients (Fig. 2A). One fifth required PPM upgrade, 13% to CRT and 8% to ICD.

**Fig. 3:**
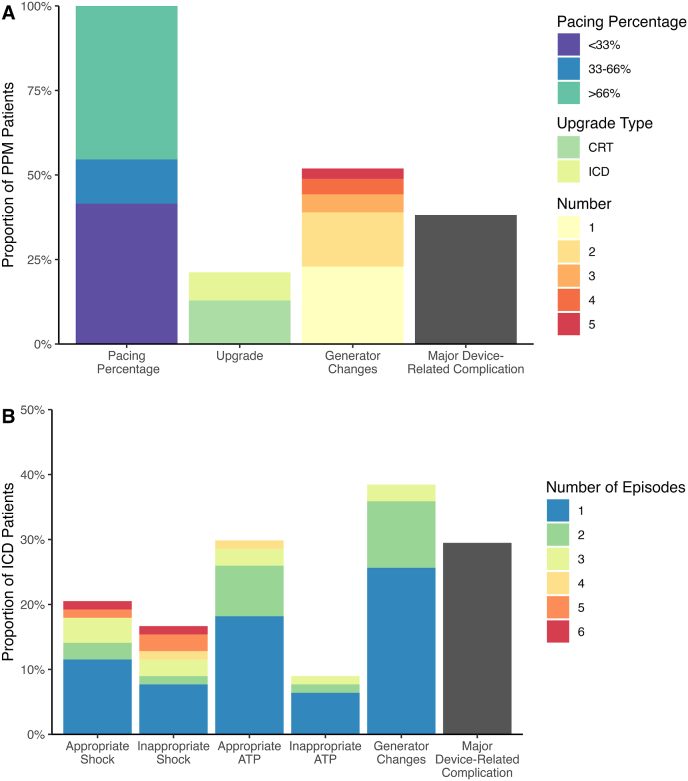
Outcome variables as a proportion of patients with (A) permanent pacemakers (PPM; n = 133); and (B) implantable cardioverter-defibrillators (ICD; n = 78).

One third of PPM patients had a device-related complication (Fig. 3A). The most common acute PPM complications were lead dysfunction requiring revision (2%), pneumothorax (2%), pleural effusion (2%) and pocket infection (2%). The most common long-term complications were lead dysfunction requiring revision (16%) and pocket infection (2%; total short/long-term infection rate: 4%). Patients with severe disease were more likely to experience a complication than those with mild disease (40% vs 27%), but this difference was non-significant (*t*-test, p = 0.42). Half of PPMs required a generator change in the follow up period.

### 3.2 ICD

Of 78 ICD patients, 47 were for primary prevention (in patients who had never had a life-threatening arrhythmia but were at high risk for one) and 31 for secondary prevention (in patients who had already experienced a life-threatening arrhythmia, in order to prevent recurrence). Primary prevention ICD patients often had multiple risk factors as indications for insertion. Non-sustained ventricular tachycardia was listed as one indication for ICD insertion in 23 primary prevention patients (49%), inducible ventricular tachycardia on electrophysiology study in 15 patients (32%), severe systemic ventricular impairment in 11 patients (23%), syncope in 9 patients (19%) and palpitations in 5 patients (11%). Overall, 39 out of 332 eligible TOF patients had an ICD (12%), 9 out of 217 TGA patients treated with an atrial switch operation (4%), 6 out of 251 with coarctation of the aorta (2%), and 5 out of 71 with Ebstein's anomaly (7%); 20 patients had other CHD diagnoses. Median age at implantation was 39 years and the cohort was 60% male.

As with PPMs, the rate of ICD insertion increased significantly with disease severity (χ^2^-test, p < 0.001; Fig. 1). Over a median 4.6 year follow up period (IQR 0.9–8.3 years), 32% of the total ICD cohort received at least one appropriate ICD intervention: 24% of primary prevention patients and 55% of secondary prevention patients (*t*-test, p = 0.006; 95% confidence interval of the difference: +9.3% to +53.5%). There were 33 appropriate shocks in 15 patients (19%) and 34 inappropriate shocks in 13 patients (17%; Fig. 3B). Inappropriate shocks were mainly caused by atrial fibrillation (AF; 30% of shocks), atrial tachycardia (26%) and T-wave oversensing (12%). Anti-tachycardia pacing was delivered appropriately for 28% of patients, and inappropriately for 9%. Inappropriate ATP was mainly precipitated by AF. All-cause mortality was 3% over the follow up period (Fig. 2B), none of which was attributable to the device itself.

Apart from inappropriate therapy, one-third of ICD patients experienced other device-related complications (Fig. 2A). Common device-related acute ICD complications included lead dysfunction requiring early revision (4%) and pocket hematoma (3%), while long-term complications were lead failure requiring revision (17%) and box repositioning due to erosion, discomfort or dysfunction (5%). Including inappropriate therapy, ICD patients faced significantly more complications than PPM patients (log-rank test, p = 0.039; Fig. 2A), though the difference fell just short of statistical significance when inappropriate therapy was excluded (p = 0.057). Patients with severe disease were more likely to experience a complication than those with mild disease (47% vs 33%), but this difference was non-significant (*t*-test, p = 0.73). In the follow up period, 40% of ICDs required at least one generator change.

## 4 Discussion

ACHD patients with implantable anti-arrhythmic devices experienced high rates of appropriate therapy, inappropriate therapy, and device-related complications. One third of PPM patients experienced a device-related complication, and 20% of PPMs required upgrade to CRT or ICD. Half of all ICD patients experienced a device-related complication or inappropriate therapy. Notably, the rate of implantable anti-arrhythmic device insertion increased significantly with disease severity.

We observed a sizeable proportion of PPM and ICD patients experiencing device-related complications. These complications ranged from acute issues such as lead positioning failure, pneumothorax and pleural effusion, to long-term concerns such as lead failure requiring revision and box repositioning. This high rate of complications replicates previous research that finds an increased risk for both PPM and ICD insertion in ACHD patients [around one third for both devices; [[9], [14]]] when compared to patients with acquired heart disease in structurally normal hearts [a range from 4% to 15% for both devices; [[15], [16], [17], [18]]], supporting our hypothesis that the risk profile of ICDs and PPMs may indeed be altered in ACHD patients. Many factors may contribute to this risk, including unique anatomical challenges to insertion [5,19], and (younger) patient age at implantation, which is an independent predictor of long-term PPM complications [14].

The complication rate in our study closely matches those found in similar retrospective studies [20] and large meta-analyses of device outcomes in ACHD [8], while studies with shorter follow up times [21] and/or those excluding epicardial lead placement [22] appear to underestimate the true complication rate over the expected lifespan of the device. In terms of mortality on the other hand, our finding of 3% for both PPMs and ICDs (none of which was attributable to the device itself) is towards the lower end of the broad range of 0.2–29.4% found in the literature, depending on the study inclusion criteria, follow up time and device under investigation [21,23]. Our inclusion criterion requiring patients to have been seen at least once since 2018, chosen to guarantee access to patient records, may have limited our potential follow up time and thus our estimate of late mortality. Nevertheless, since the RPAH ACHD database maintains up-to-date mortality statistics via linkage with the Australian NDI, we can be confident that no deaths in this period were missed.

Our paper found a high rate of inappropriate ICD shocks compared to appropriate shocks. This is in keeping in previous literature that suggests that CHD patients with ICDs experience inappropriate shocks at a rate roughly twice that of non-CHD patients [[24], [25], [26], [27]]. Appropriate shocks, in this study and others, match those found in patients with acquired heart disease [27]. This suggests that CHD patients suffer from a genuine predisposition to inappropriate shocks, rather than simply a low shock threshold. As in previous literature, this study found that inappropriate ICD shocks were mainly triggered by atrial arrhythmias [28], to which ACHD patients are strongly predisposed [29]. Device programming can be difficult in this setting, for example in the context of 1:1 conduction of intra-atrial re-entrant tachycardia exceeding the specified rate limit for supraventricular tachycardia discrimination. It is plausible that the unique anatomical challenges posed by CHD in terms of device insertion and functionality may also contribute [5], while younger age and more active lifestyle may play a role in inappropriate shocks triggered by sinus tachycardia [19]. Given the significant psychological and clinical impact of inappropriate shocks [27,30], our findings underscore the importance of careful device programming and consistent patient follow up. This is especially true in patients with severe disease, who we found to be much more likely to receive an ICD or PPM, replicating and extending previous research on PPMs specifically [14].

Due to the unique centralisation of Australian ACHD care at one quaternary centre per state, we chose to use the comprehensive RPAH ACHD database to examine a true cross-section of CHD patients across the lifespan and across the spectrum of disease severity. Universal insurance under Medicare improves the validity of an Australian-based cohort, compared to other jurisdictions where insurance status might govern access to expensive devices such as these. Due to the rarity of many CHDs, however, even the large RPAH ACHD database cannot produce a sample size large enough to publish statistically significant ICD and PPM results on a lesion-by-lesion basis. Fortunately, our research group is responsible for the construction of the National Registry of the Congenital Heart Alliance of Australia and New Zealand (CHAANZ), which will unify clinical data from all CHD patients in Australia and New Zealand, and allow us to corroborate the results of this study in a much larger cohort and examine the outcomes of ICDs and PPMs in individual congenital heart lesions. It is our hope that the CHAANZ Registry, which will contain the results of individual interviews with a subset of CHD patients, will also allow us to examine the significant but understudied psychological burden of arrhythmia in CHD, alongside the evident clinical burden.

## 5 Conclusions

Anti-arrhythmic devices can be lifesaving in ACHD patients, but inappropriate therapy and device-related complications are very common, highlighting the need for implantation and follow up in experienced centres.

## Sources of funding

Nil relevant.

## Grant support

Nil relevant.

## Sources of funding

Nil relevant.

## CRediT authorship contribution statement

**Jason Chami:** Conceptualization, Data curation, Formal analysis, Investigation, Methodology, Resources, Validation, Visualization, Writing - original draft, Writing - review & editing. **Benjamin M. Moore:** Data curation, Supervision. **Calum Nicholson:** Investigation, Methodology, Project administration, Software. **Rachael Cordina:** Data curation, Project administration, Resources. **David Baker:** Supervision, Validation. **David S. Celermajer:** Conceptualization, Data curation, Project administration, Resources, Supervision, Writing - review & editing.

## Declaration of competing interest

The authors declare the following financial interests/personal relationships which may be considered as potential competing interests:Co-author serves on the editorial board of IJCCHD - D.C.
